# Smoking in Teenagers from the Social Protection System—What Do We Know about It?

**DOI:** 10.3390/medicina57050484

**Published:** 2021-05-12

**Authors:** Corina Eugenia Budin, Ruxandra-Mioara Râjnoveanu, Ioana Roxana Bordea, Bianca Liana Grigorescu, Doina Adina Todea

**Affiliations:** 1 Department of Pathophysiology, University of Medicine, Pharmacy, Science and Technology Târgu Mureș, 540139 Târgu Mureș, Romania; cora_bud@yahoo.com (C.E.B.); biancagrigorescu20@yahoo.com (B.L.G.); 2 Department of Pneumology, University of Medicine and Pharmacy “Iuliu Hațieganu” Cluj Napoca, 400012 Cluj Napoca, Romania; andra_redro@yahoo.com (R.-M.R.); doina_adina@yahoo.com (D.A.T.); 3 Department of Oral Rehabilitation, University of Medicine and Pharmacy “Iuliu Hațieganu” Cluj Napoca, 400012 Cluj Napoca, Romania

**Keywords:** smoking, foster care, teenagers, social protection

## Abstract

*Background and Objectives*: The impact of smoking on the young population is an extremely important issue for the public health system. As the prevalence of smoking is considerably increasing amongst the pubescent and adolescent population, the prevention of smoking at this age should be considered of high priority. The primary aim of this observational study was to assess tobacco use in teenagers included in the social protection system. *Materials and Methods*: 275 foster care teenagers (155 from the Professional Maternal Assistance System (AMP) and 120 from the residential system) from two different counties were enrolled. After a brief interactive session focused on the main consequences of smoking, a self-administered questionnaire was anonymously completed. *Results*: The mean age of the study group was 14 years, with a significant difference between the residential system and AMP (*p* = 0.001). Smoking status was significantly higher in participants from family-type houses (36.7%) than in those from the AMP (11.7%) (*p* < 0.001). The presence of smokers in the family (78.3%) and passive smoking (64.7%) were significantly higher in children from the residential system than in those from the AMP (32.9% and 31.8%, respectively) (*p* < 0.001). The number of cigarettes consumed daily was associated with the age of the participants (*p* = 0.01, rho 0.42). In total, 82.3% were cigarette users and 19.4% were e-cigarette users. Smokers bought the majority of their cigarettes from the store (63.2%) or asked a friend (19.3%) or an adult to buy them on their behalf (12.3%). *Conclusions*: The null hypothesis, according to which children who are abandoned but raised and cared for by professional nursing assistants are predisposed to earlier tobacco activity compared to children raised in a normal familiar environment, is supported. Future education and prevention campaigns conveying the benefits of a healthy long-term lifestyle to this population category are needed.

## 1. Introduction

The prevalence of smoking in the young population is increasing, especially among pubescent and adolescent individuals; therefore, the prevention of smoking at this age should be considered a priority for the public health system [1]. At least 1 in 10 adolescents aged between 13 and 15 years old smokes, although there are places where the onset of smoking among children starts at a younger age. Reviews indicate that well-designed and implemented social programs and interventions can effectively help adolescents quit smoking [2] and prevent them from using other substances, such as alcohol or drugs [3,4,5].

Prohibition of the sale of tobacco products to minors, price increases, warnings on cigarette packets, and provision of smoke-free spaces are crucial in preventing smoking in teenagers (Law No. 15/2016 to prevent and combat the consumption of tobacco products). In Romania, it is forbidden to sell tobacco products to minors. Although the law provides for these prohibitions, underage smokers still find ways to obtain tobacco products.

Worldwide, there are 143 million children separated from their families, and of these, approximately 95% are institutionalized in the Maternal Assistance system [5]. In early 1990s, Romania inherited a precarious child protection system, in which approximately 100,000 children were institutionalized in large placement centers. It has been proven that, over time, the residential system has detrimental effects on the physical and mental development of minors. Consequently, in the last 25 years, remarkable progress has been made in the Romanian social protection system of children, with a drastic reduction in large placement centers and an increase in the number of children in the Maternal Care system. The current trend is to place children as early as possible into the Maternal Care system. In the social protection system, children can be included from birth, and can remain there until the age of 26 if they continue their studies [6].

In Romania, law No. 272/2004 on the protection and promotion of the rights of the child, republished with subsequent amendments and additions, lays down both types of services intended to prevent the separation of children from their parents and those for the special protection of children that are temporarily or permanently separated from their parents. The following types of services were organized and offered: day services, family-type services, and residential services.

Day services include day centers; counselling and support centers for parents; assistance and support centers for the rehabilitation of children with mental and social problems; and monitoring, assistance, and support services for pregnant women who are likely to abandon their children.

The role of residential services is to ensure the protection, upbringing, and care of children temporarily or permanently separated from their parents, following the terms of the placement measure law. These services include foster homes (including family-type homes), emergency child reception centers, and foster care centers.

The Maternal Assistance Service is responsible for ensuring, for a specified period of time, the raising and care of children temporarily or permanently separated from their parents, in accordance with the protective measure law. The Maternal Assistance Service operates in compliance with the provisions of the general framework for the organization and functioning of social services governed by Law No. 292/2011, as an amendment of Law No. 272/2004 on the protection and promotion of the rights of children that was republished, with subsequent amendments and additions, as well as other secondary regulatory acts applicable to the field [5,6,7].

According to the 2015 data from the National Institute of Statistics in Romania, there are 57,279 teenagers under the child protection system. Among them, 20,291 are in state residential services (foster care homes) and 36,980 in family care (children with social parents). The impact of the Romanian child protection system reform is evident, as the annual statistics show a significant decrease in the number of children benefiting from the special protection measures in the residential services and in the number of these residential institutions. The 2016 national statistics also revealed that 66% of minors are institutionalized under family-type services (34,300 children), of which 34.0% are in foster care, 25.0% are placed with relatives, and 7.0% are placed with acquaintances. Furthermore, 17.0% (9000 children) are institutionalized in small-sized residential care as follows: 4.0% in flat residence, 4.0% in family homes equipped with facilities for disabled children, and 9.0% in family homes for children without disabilities. In addition, 17.0% (9000 children) are in transition centers [7].

There is a large age variability among people in the child protection system in Romania, ranging from 0 to 26 years old, with 56.0% of them aged between 10 and 17 years old. Regarding gender and environment distribution, 53.0% are males and 47.0% females, while 43.0% originate from rural areas and 56% from urban areas. The distribution of ethnicity among these children is significantly different from the young general population. The proportion of Roma children under protection (10.3%) is double that of the general young Roma population (5.3%). Similarly, Romanian children under protection represent 54.0%, compared to 79.1% in the general young population. Children with a disability represent a significant proportion of all children in the care system (29.0%). Of those, 6.0% are below the age of one year old and 43.0% are between 18 to 26 years old or older [7,8,9]. Institutionalized minors, along with adolescents with behavioral and emotional problems, as well as those with nontraditional sexual orientations, belong to these vulnerable categories. After separation from the biological family, these young people experience additional risks, such as placement instability, multiple changes of schools, or a lack of affection from foster carers. All of these negative experiences increase the risk of smoking initiation at younger ages [4,5].

The present study aimed to assess the smoking status and the level of knowledge about tobacco use in minors from the social protection system. The null hypothesis of the study was that foster care minors are predisposed to early onset of smoking compared to those from a traditional family environment. The alternative hypothesis was that foster care minors initiate smoking in a similar manner to that of the general population.

## 2. Materials and Methods

This observational, transversal study took place between 15 November, 2016 and 15 December, 2018, and was carried out in the general population represented by all institutionalized minors aged between 10–18 years from Mureş and Cluj counties. Being a general population study, all adolescents from the database of the General Direction of Child Protection who agreed to participate were included. The participation rate was 75.34% (275 persons from 365 registered in the database of the General Direction of Youth Protection).

Since the participants were minors, the written informed consent was also signed by their tutors, represented by the foster professional or by the General Directorate of the Social Assistance and Child Protection manager. The study received the approval No. 443/Nov 2016 from the Ethics Committee of the “Iuliu Hatieganu” University of Medicine and Pharmacy Cluj-Napoca, Romania, respecting the standards of personal data protection.

The minors from the social protection system enrolled in the study originated from the Professional Maternal Assistance System (AMP) and from the residential system, including those from family-type homes and day shelters, respectively. Participants were divided into groups of 20–25 participants. For each of these groups, the study was conducted in the morning of a single day in order to have more focused and rested participants. The meeting session started with a 20 min video material about the effects of tobacco use followed by an interactive discussion. The purpose of this presentation was to inform the children about the harmful effects of smoking because they have never participated in educational projects on this topic. Following the video presentation, we did not aim for short-term feedback at the end of the presentation; the goal was purely educational.

At the end of this session, the participants anonymously filled in a questionnaire.

The video was unable to influence the data in the questionnaire, as the presentation contained general data about smoking and its harmful effects, and the questionnaire consisted of personal questions related to each participant and did not assess the degree of knowledge related to smoking.

The questionnaire was self-reported and included 31 questions (13 single-choice questions, 15 multiple-choice questions, and 3 open-ended questions) regarding demographic characteristics, tobacco-use-related variables (the number of cigarettes consumed daily, degree of physical dependence on nicotine, knowledge on tobacco legislation, and second-hand exposure to cigarette smoke), and teenagers’ perception of smoking. Twenty-three questions were about cigarette smoking and eight questions about e-cigarettes. (Supplementary File S1). The questionnaire was prepared in collaboration with a specialized sociologist and included all items deemed to be necessary and sufficient for the purpose of this study. The maternal assistants (foster parents) were present in the room during the video presentation, but when the questionnaires had to be filled out, they left the room so that the teenagers completed the survey without being influenced by their presence. Before the completion of the questionnaires, the subjects were instructed on the voluntary nature of the test. The questionnaires were collected collectively at the headquarters by the team of researchers without any representation of the institution’s members.

A current smoker was defined as someone who had smoked more than 100 cigarettes (including hand rolled cigarettes, cigars, cigarillos) in their lifetime and had smoked in the last 28 days. An ex-smoker was defined as someone who had smoked more than 100 cigarettes in their lifetime but had not smoked at all in the last 28 days. A never smoker was defined as someone who had not smoked more than 100 cigarettes in their lifetime and currently does not smoke [10].

Definitions of intermittent smoker or light smoker are usually used for teenagers, and there are similar to low-level daily or low-rate daily smokers [10,11]. This classification was also used for the teenagers in our study.

According to the World Health Organization, adolescence is the phase of life between childhood and adulthood, from age 10 to 19. It is a unique stage of human development and an important time for laying the foundations for good health.

A “minor” can mean a person with an age below that of the majority. In Romania, the age of the majority is 18 years old or above.

The United Nations Convention on the Rights of the Child (UNCRC) defines a “child” as an individual under 18 years old, and, unless under the law applicable to children, the majority is defined as mentioned above.

### Statistical Analysis

The Microsoft Excel program was used to centralize the data. The statistical analysis was carried out in R (v. 3.4.4, cran.r-project.org (accessed on 25 march 2018)). The participants’ characteristics are expressed as the median and inter-quartile range for continuous variables (after the observation that they had non-Gaussian distributions) and by absolute number and percentages for categorical variables. For statistical inference, parametric or nonparametric methods were used, depending on the distribution of the variable values. The threshold of statistical significance was 0.05. Data from minors in professional foster care system were also processed using SPSS software (IBM SPSS Statistics 25.0.0.0, Armonk, New York, NY, USA).

## 3. Results

The study group consisted of 275 participants, 155 from the Maternal Assistance System (AMP) and 120 from the residential system (family-type homes). The general data of the study participants are presented in Table 1. The gender distribution was not significantly different between the AMP and residential System (*p* = 0.997). The mean age of the study group was 14 years, with significant differences between the residential system and AMP (*p* = 0.001).

A proportion of 22.6% (62) of the participants were smokers, 18 (29%) were from the AMP and 44 (71%) were from the residential system. Smoking status was significantly higher in participants from family-type houses (36.7%) compared to the AMP (11.7%) (*p* < 0.001). The average age of smokers was 15 years (14.0;17.0). Those in the residential system had an average age of 16 years, which was higher than those in the AMP system (14.5 years) (*p* = 0.139). The presence of smokers in their family was significantly higher in children from the residential system (78.3%) than that of those in the AMP (32.9%) (*p* < 0.001) (for the teenagers in the residential system, the family is represented by the family type-house).

A total of 124 children reported being exposed to passive smoking. Those included in the residential system (64.7%) were significantly more exposed to second-hand smoke than those from the AMP (31.8%) (*p* < 0.001).

According to the frequency of smoking in the smoker group, 16 (27.6%) declared that they were occasional smokers, with 8 (50%) in the AMP and 8 (19%) in the residential system. The proportion of daily smokers in family homes (81%) was higher than that in the AMP (50%), as presented in Table 2. The proportion of daily smokers by gender is found in Table 3.

The estimated age for the consumption of the first cigarette in all participants was 12 years of age. The estimated age for the first cigarette in AMP participants was 12.5 years of age and in the residential system 11.5 years of age. The distribution by age group of smoking onset is described in Table 4.

**Figure 1:**
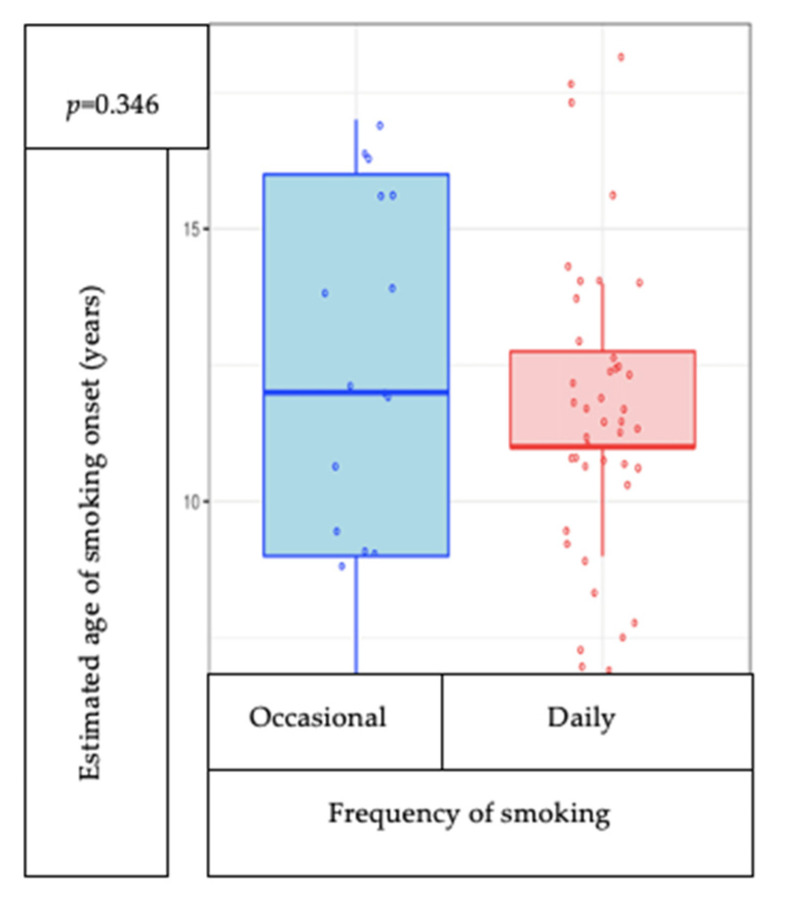
Correlation between declared age of smoking onset and frequency of smoking.

**Figure 2:**
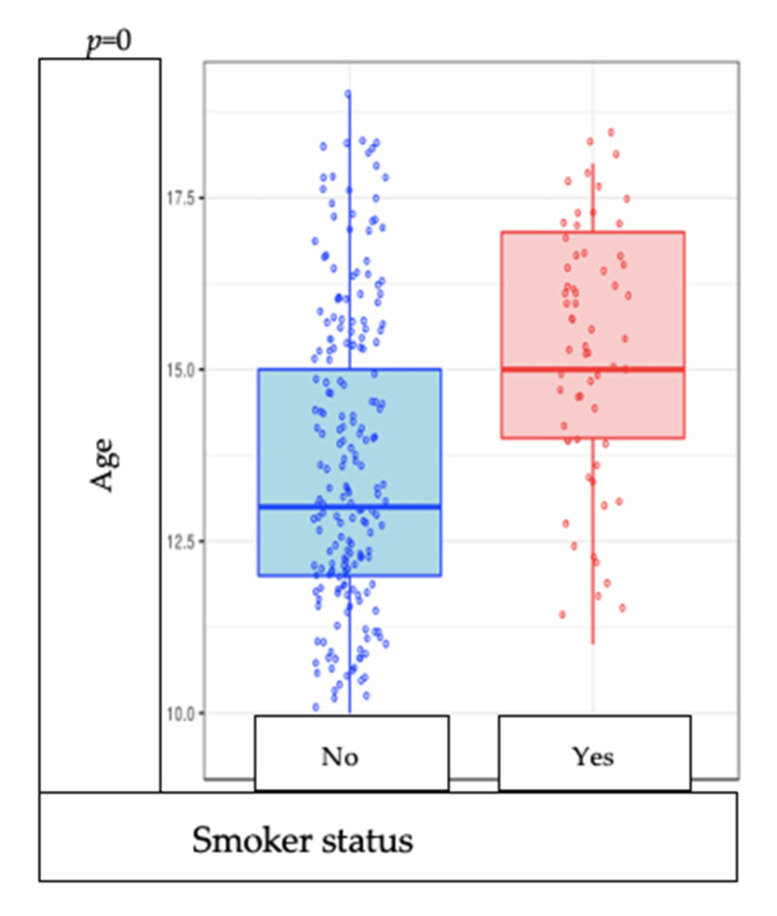
Correlation between age and smoking status.

The estimated age of onset of smoking is not correlated with the frequency of smoking (*p* = 0.346) (Figure 1). Of the whole group of participants, smokers are significantly older than nonsmokers (*p* = 0.000) (Figure 2).

**Figure 3:**
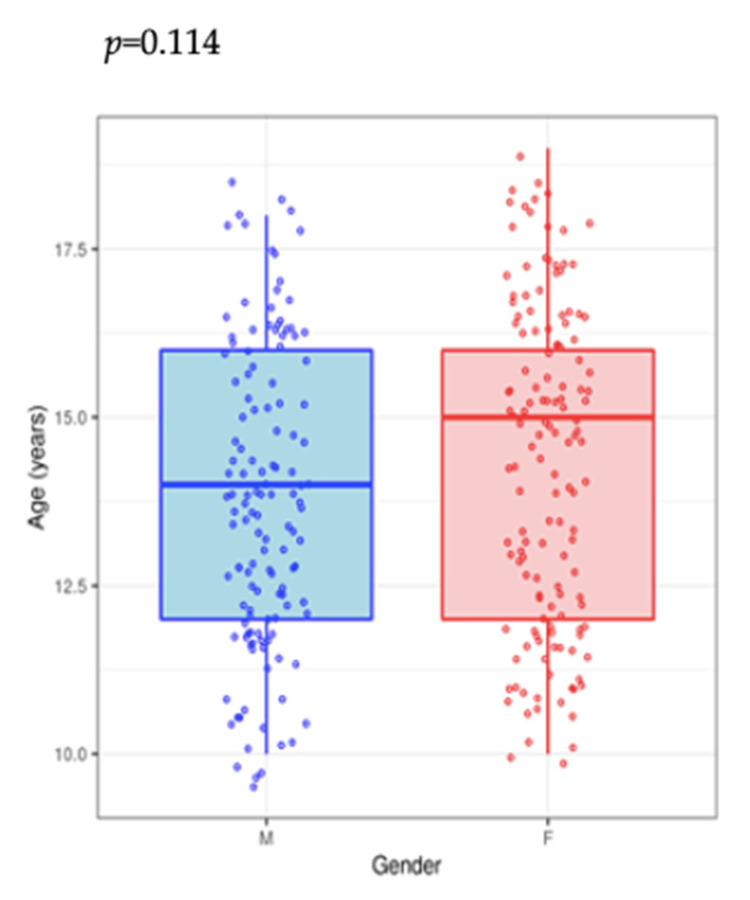
Correlation between age and gender in the study group.

**Figure 4:**
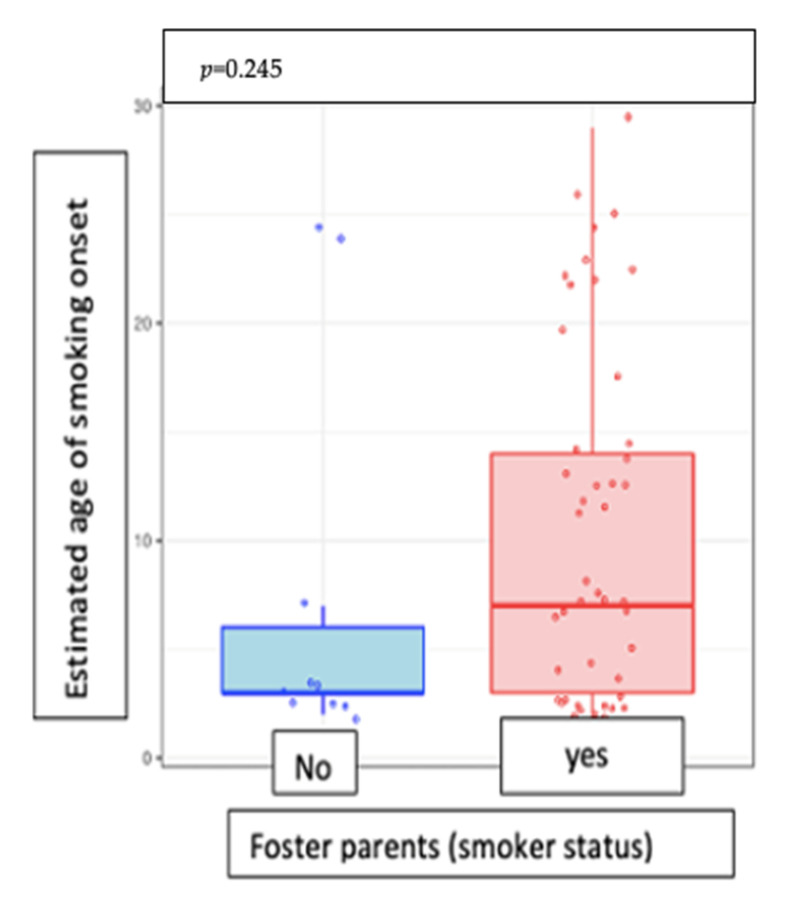
Correlation between age of smoking onset and smoker status of foster parents.

There was no significant sex difference in terms of the smoking onset age or in the number of cigarettes consumed daily (*p* = 0.114) (Figure 3). Moreover, the estimated age of smoking onset was not associated with smoking in foster care (*p* = 0.245) (Figure 4).

**Figure 5:**
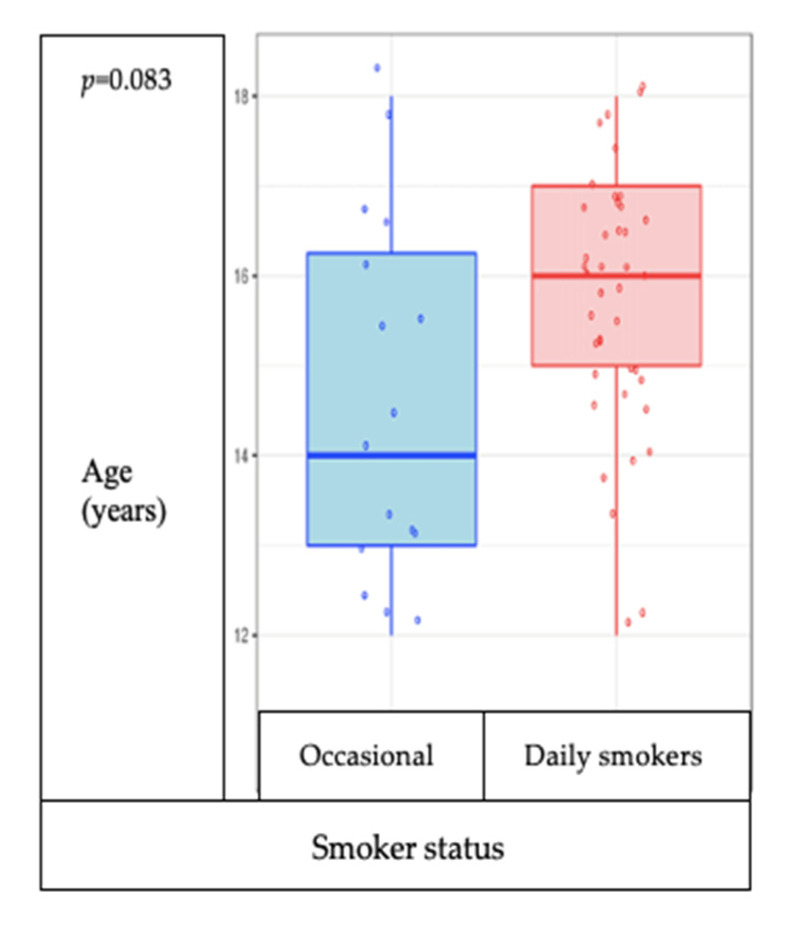
Correlation between age and frequency of smoking (daily and occasional smoker groups).

**Figure 6:**
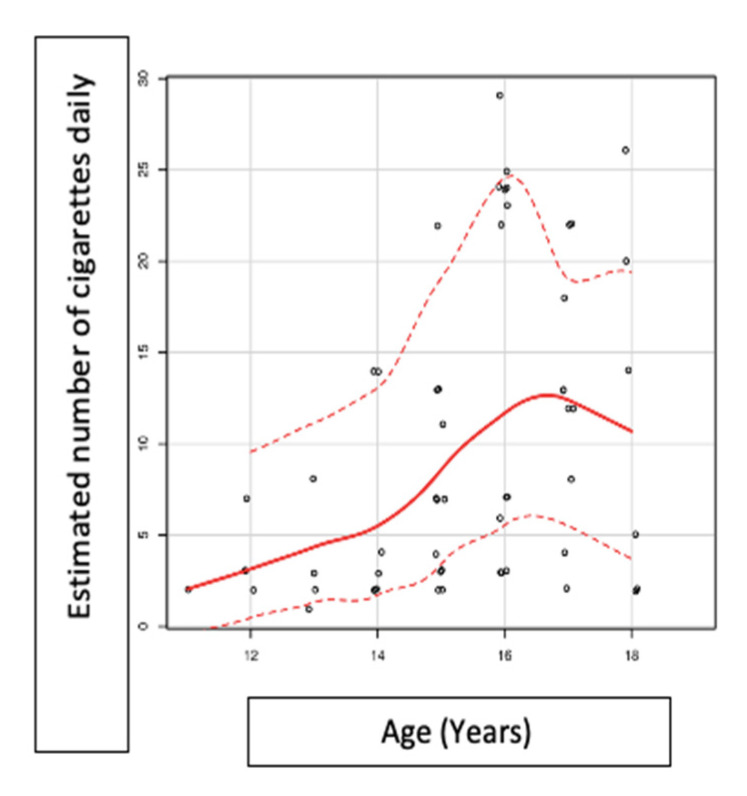
Correlation between age and the number of daily consumed cigarettes.

Study participants from the residential environment initiate smoking at a younger age than those in the AMP system and have a pronounced tendency to smoke more cigarettes per day. In the study group, there is no association between the age of the participants and the frequency of smoking (occasional smokers/daily smokers) (*p* = 0.083), but the tendency is for those older to smoke daily and not occasionally (Figure 5), and the number of cigarettes consumed daily is associated with the age of the participants (*p* = 0.01, rho 0.42) (Figure 6).

After the assessment of the questionnaire replies, some particular aspects were identified. The average number of estimated cigarettes/day was 7.00 [3.00;14.0]. Regarding the type of the tobacco products consumed, 82.3% of the participants stated that they were cigarette users, 6.45% smoked rolled cigarettes, 9.68% smoked cigars, and 19.4% were e-cigarette users. Unfortunately, we do not have data on mixed tobacco use and e-cigarette use, as only a simple question in relation to this was asked: did you use e-cigarettes? We do not have data on the period of use.

Smokers bought most of their cigarettes from the store (63.2%), asked a friend (19.3%) or an adult to buy them on their behalf (12.3%), or stole them from their caretakers (5.26%).

The preferred sources of smoking information were school (59.7%), entourage (50%), and family (40.3%).

The reasons for smoking were the following (in order of the number of answers): entourage (38.7%), relaxation (35.5%), smoking “gives me energy” (16.1%), gesture (12.9%), habit (11.3%), addiction (8.06%), curiosity (6.45%), others (6.45%), “do not know why” (6.45%), fun (4.84%), boredom (4.84%), and confidence (4.84%) (Table 5).

Another question asked was about the effects that smoking cessation might cause, and the answers are analyzed in Table 6.

Of those who have been exposed to secondhand smoke, the majority (58.1%) stated that they were exposed to passive smoking at school, 35.5% in the family home, and 3.23% in public spaces.

## 4. Discussion

In institutionalized children, increased percentages of smokers have been documented in the literature compared to the values of national groups not included in the social protection system [12,13,14]. A study by Braciszewskia and Colby showed that the percentage of smokers among minors in the social protection system is almost three times that of the national value [13,14].

Compared with these results from Europe and the USA, in our study, the frequency of smoking in minors aged between 10–18 years from the social protection system was approximately double that of the national group. Thus, the results of our research show that there are 22.6% more smokers among the respondents when compared to the latest official survey conducted by the World Health Organization in children not included in the social protection system (Global Youth Tobacco Survey, GYTS Romania), which provided the following results: 11.2% current consumption of tobacco products for both sexes (12.2% males and 10.1% females), and for daily cigarette smoking, a percentage of 9.4% (10.1% for males and 8.5% for females) [8,15,16]. The World Health Organization included young people over the age of 15 in the Global Adult Survey, and the results for daily smokers were 24.3% for both sexes (34.9% for the male population and 14.5% for the female population), [1,16,17]. These results need to be adapted because there are differences between the age groups of the two studies (GYTS: 13–15 years; our study: 10–18 years). Although the two groups (those in GYTS and that in our study) are not completely superposable, the conclusion was that in our study, the frequency of smoking was higher than that in the general population.

The earlier young people start using alcohol or tobacco, the greater their vulnerability to developing an addiction to such substances or to illicit drug use [18,19]. Therefore, effective smoking prevention can be beneficial for controlling other addictions [4,13,18,20,21,22].

Adolescence, the period of transition to adulthood, is a decisive period for young people in the social protection system. Teenagers in foster care do not benefit from family support like those in the general population. Therefore, the authorities ask foster carers to behave like parents, because it has been proven that this is beneficial to teenagers. There are educational programs that encourage friendly relationships between adults and institutionalized minors. Those who manage to be adopted have an increased chance of becoming adults with a normal social life [23,24].

For the Transylvania region, of which Mures and Cluj counties are a part, a cross-sectional study conducted in four counties, which included 914 institutionalized minors in a residential system, showed that 62% of participants declared themselves to be smokers or former smokers, and 46% as recent consumers of tobacco products (at least once in the last three months) [24,25]. This was the only study conducted before our research on the frequency of smoking in minors institutionalized in Romania. In that study, 32% identified as daily smokers, while in our study, 22.6% of the participants reported daily smoking.

Compared to the data from GYTS Romania (35.5% of students were exposed to second-hand smoke at home), in our study, the overall percentage of exposure to passive smoke was higher (46.4%), with the percentage differences depending on the environment of origin, in that in residential areas, the percentages were much higher (68.2% compared to 35.3%) [8,15,16].

Exposure to second-hand smoke is higher in family-type homes (32.9%) compared with that in the Professional Maternal Assistance System (22.2%), once again confirming the above-mentioned hypotheses. However, the most significant exposure to second-hand smoke was declared by respondents to be in school, which justifies the initiation and continuation of educational programs to prevent and combat smoking in schools. This conclusion is also supported by the correlation of our study between the number of cigarettes consumed daily and age. Although the correlation is highly statistically significant (*p* = 0.001, rho = 0.42), the association is closest until around the age of 16–17, thus signifying that, after this age, young people become more aware of health risks. Therefore, smoking education programs should be started at an early age, especially in schools, as it is the source of information that most respondents preferred [26,27].

The limitations of this survey are connected to the confidence of the respondents due to the self-reported nature of the questionnaire. The respondents may have also underreported smoking behavior due to their young age. Thus, we were unable to obtain data related to the socio-economic level or family situations and dynamics.

Another limitation is that we did not evaluate the impact of the video presentation on the teenagers, so we did not have a before–after evaluation measuring the change in people’s knowledge, attitudes, and intentions regarding tobacco use.

As only two counties were included, the relatively small number of subjects can be another limitation of the study. Fortunately, the number of institutionalized minors has decreased in recent years.

The applicability of the study is based on the observations that teenagers in foster care admitted to having started tobacco use and addictive behavior at an early age. As a result, the preventive programs should start early, and they should provide adequate conditions to living environments. The need to prevent or delay the use of tobacco should be emphasized, since smoking could be a gateway to other drugs [14,28].

## 5. Conclusions

The null hypothesis, according to which children who were abandoned but raised and cared for by professional nursing assistants are predisposed to earlier tobacco activity compared to children raised in a normal familiar environment, is demonstrated. Furthermore, teenagers from the residential system are more exposed to active and passive smoking than those from the Professional Maternal Assistance System. Building on the premise that nicotine addiction is a combination of pharmacological, genetic, and environmental factors, our research is a pilot project that can chart important directions in future education and prevention campaigns, bringing benefits to this population category for a healthy long-term lifestyle.

## Figures and Tables

**Table 1:** General data of the study participants.

	Total	AMP	Residential	*p* Overall
General data	*N* = 275	*N* = 155	*N* = 120	
Age ^1^	14.0(12.0;16.0)	13.0(12.0;15.0)	15.0(12.0;16.0)	0.001
Gender:				0.997
Male	133 (48.7%)	75 (48.4%)	58 (49.2%)	
Female	140 (51.3%)	80 (51.6%)	60 (50.8%)	
Smoking status:				<0.001
Smoker	62 (22.6%)	18 (11.7%)	44 (36.7%)	
Ex-smoker	12 (4.38%)	5 (3.25%)	7 (5.83%)	
Nonsmoker	200 (73.0%)	131 (85.1%)	69 (57.5%)	
Smokers in the family				<0.001
No	130 (47.3%)	104 (67.1%)	26 (21.7%)	
Yes	145 (52.7%)	51 (32.9%)	94 (78.3%)	
Global exposure at passive smoking				<0.001
Yes	124 (46.4%)	47 (31.8%)	77 (64.7%)	
No	143 (53.6%)	101 (68.2%)	42 (35.3%)	

^1^ Comments: Age, as a continuous variable, has no Gaussian distribution; the comparison test is nonparametric, and the values in the table are median and the inter-quartile range of 25–75%. The categorical variables were compared with the chi-squared test; the number and percentage of participants for each variable are displayed. The missing values up to the total are nonresponses.

**Table 2:** Frequency of smoking (smoker group).

Frequency	Total	AMP	Residential
Occasional	16 (27.6%)	8 (50.0%)	8 (19.0%)
Daily	42 (72.4%)	8 (50.0%)	34 (81.0%)

**Table 3:** Daily smoker analysis *.

	Male (*n*, % **)	Female (*n*, %)	Total (*n*)
AMP	5 (11.9%)	3 (7.2%)	8
Residential	21 (50%)	13 (30.9%)	34

* The missing values up to that total are nonresponses. ** % from all daily smokers.

**Table 4:** Estimated age for the first-cigarette categories.

	Total	AMP	Residential	*p* Overall
Age for the first-cigarette categories:	*N*, %	*N*, %	*N*, %	0.349
<8 years	6 (9.68%)	1 (5.56%)	5 (11.4%)	
8–10 years	11 (17.7%)	2 (11.1%)	9 (20.5%)	
11–12 years	26 (41.9%)	6 (33.3%)	20 (45.5%)	
13–14 years	10 (16.1%)	5 (27.8%)	5 (11.4%)	
>15 years	9 (14.5%)	4 (22.2%)	5 (11.4%)	

**Table 5:** What is your main reason for smoking? *.

Answer	Valid%
Smoking gives me more energy	16.1
Gesture to smoke	12.9
Fun	4.84
Relaxation	35.5
I don’t know why	6.45
Curiosity	6.45
Entourage	38.7
Habit	11.3
Addiction	8.06
Others	6.45
Boredom	4.84
Confidence	4.84
Desire to join group	26.3

* Participants had multiple answers.

**Table 6:** Responses about quitting smoking *.

Do You Think that Quitting Smoking Would Generate Effects?					
Do You Smoke?		Negative	Positive	I Do Not Know	Total
Yes = smoker (at least 6 months)	% within: Smoke?	14.3%	14.3%	71.4%	100.0%
	% within: believe that quitting smoking would generate effects?	5.6%	2.1%	9.4%	5.9%
Nonsmoker (less than 100 cigarettes)	% within: Smoke?	14.8%	42.6%	42.6%	100.0%
	% within: believe that quitting smoking would generate effects?	89.9%	95.8%	86.8%	90.8%
Former smoker	% within: Smoke?	25.0%	25.0%	50.0%	100.0%
	% within: believe that quitting smoking would generate effects?	5.6%	2.1%	3.8%	3.4%

* Data were processed with SPSS software (IBM SPSS Statistics 25.0.0.0, Armonk, New York, NY, USA). We made a cross-tabulation between the following questions: “Do you think that quitting smoking would generate effects?” and “Do you smoke?”.

## Supplementary Materials

The following are available online at https://www.mdpi.com/article/10.3390/medicina57050484/s1, File S1: Questionnaire.
