# Supplementary material for: Smoking in Teenagers from the Social Protection System—What Do We Know about It?
Source: Medicina (Kaunas). 2021 May 12;57(5):484. doi: 10.3390/medicina57050484 (PMC8150939; doi:10.3390/medicina57050484)
Supplement: Supplementary file 1 [file medicina-57-00484-s001.zip › medicina-1173614-SI.pdf]

# Questionnaire

1. **How old are you?**
2. **What is your gender? Female/Male**
   - F
   - M

**3. Are you a smoker? Yes/No**

- **Yes** (for at least 6 months ) = smoker)
- **No** (≤100 cigarettes = non smoker)
- **Former smoker**

**4. If you answered YES at question 2:**

- Daily Smoker
- Occasional Smoker

**5. How many cigarettes do you smoke daily?**

- Less than 5
- 6 – 9
- 10 – 19
- Over 20

**6.When did you try your first cigarette ?**

- I had never smoke
- Before 8 years
- 8/10 years
- 10/12 years
- 12/14 years
- 15 years old or later

**7.What is the way you purchased your cigarettes/ tobacco products?**

- I bought them from the store,  supermarket
- I asked someone older to buy my cigarettes
- I got / asked from a friend / acquaintance
- I took them from parents or older siblings without knowing them

**8. Does your parents know that you are smoking ?**

- Yes
- No

**9. Are they agree with smoking ?**

- Yes
- No

**10. Who is smoking in your foster family ?**

- Foster mother
- Foster father

**11. What is your main reason for smoking ?**

- Smoking gives me more energy
- The pleasure of smoking
- People around me are smoking / The desire to join the group
- Smoking helps me relax
- I do not know
- Out of habbit
- Because I feel the need to smoke / I am addicted
- Entertainment / Fun
- Curiosity
- Smoking  gives me courage / trust
- Other ......................................................................................................

**12. What prompted you to start smoking ?**

……………………………………………………………………………………………………

**13. Do you think smoking helps teens feel more attractive ?**

- If yes,why?
- If no,why?

**14. Have you tried to quit smoking?**

- Yes
- No

**15. Do you think smoking cessation would have effects ...?**

- Negative
- Positive
- I do not know

**16. Do you think smoking is bad for your health?**

- Yes
- No

**17.What are the health risks associated with smoking?**

- Different forms of neoplasia
- respiratory diseases
- heart diseases
- There are no risks

**18. What sources tell you about the harmfulness of tobacco??**

- family
- school
- media (radio, TV, newspapers)

**19. Who would be the best able to convince you to stop smoking?**

- doctor
- family member
- teachers
- friends
- a religious leader
- a government official

**20. Will the recent legislative measures reduce smoking?**

- YES
- NO
- I do not know

**21.Are you exposed at passive smoking ?**

- In the family
- At school
- entourage
- not at all

**22. Do you think smoking affects the health of others (who do not smoke)?**

- yes
- no

**23. What types of cigarettes do you ussualy smoke?**

- Cigarettes
- Hand rolled cigarettes
- Cigars
- Electronic cigarette

**Regarding e-cigarette, please answer the following questions:**

**1. How did you first find out about the electronic cigarette?**

- Internet
- friends
- direct distributors
- parents
- others

**2. Have you used the electronic cigarette even if you are not smoker?**

- yes
- no
- others

**3. If no,do you want to try it ?**

- Yes
- No

**4. Do you think e-cigarette is dangerous for your health?**

- yes
- no
- I do not know

**5. What caused you to use it?**

- curiosity
- entourage
- Others

**6. How long have you been using it?**

**..............................................................**

**7. Where do you use it?**

- At home
- At school
- At the party
- others

**8. How did you buy it?**

- Internet
- Entourage
- others
